# Supplementary material for: Health Catch-UP!: a realist evaluation of an innovative multi-disease screening and vaccination tool in UK primary care for at-risk migrant patients
Source: BMC Med. 2024 Oct 29;22:497. doi: 10.1186/s12916-024-03713-4 (PMC11520889; doi:10.1186/s12916-024-03713-4)
Supplement: Supplementary file 1 — Supplementary Material 1: Figure S1. MRC Framework on Complex Interventions. Figure S2. Health Catch-UP! Screening and Catch-up Vaccination Prompts. Figure S3. Health Catch-Up! Care Pathway. [file 12916_2024_3713_MOESM1_ESM.docx]

**Supplementary Files**

**Supplemental Figure 1: MRC Framework on Complex Interventions**


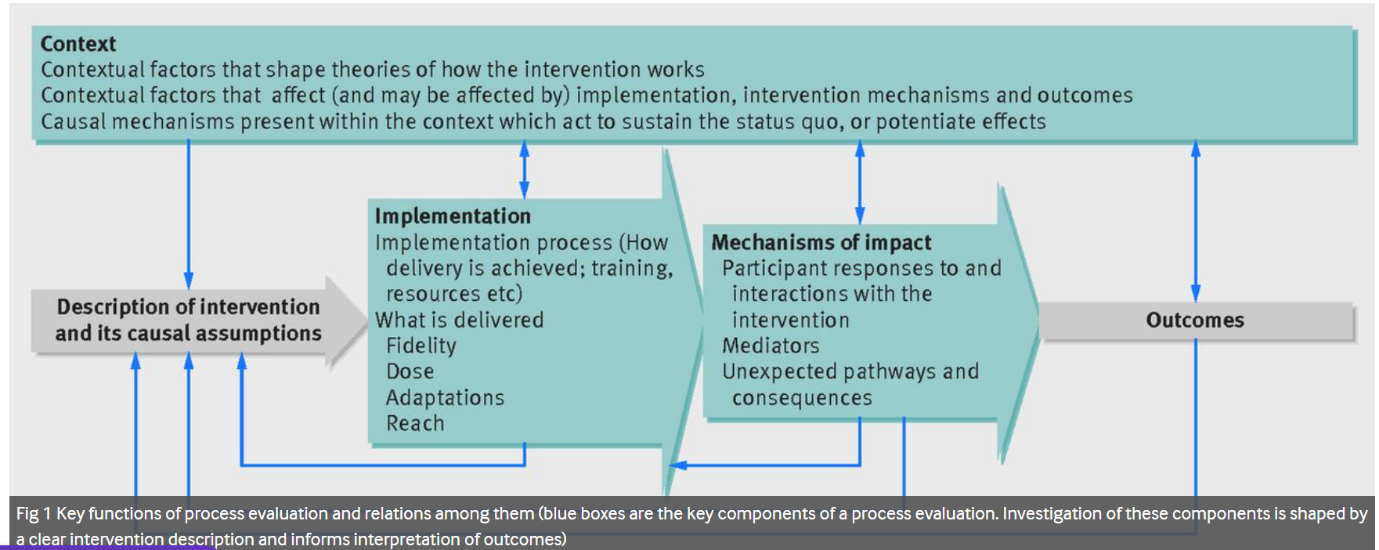


**
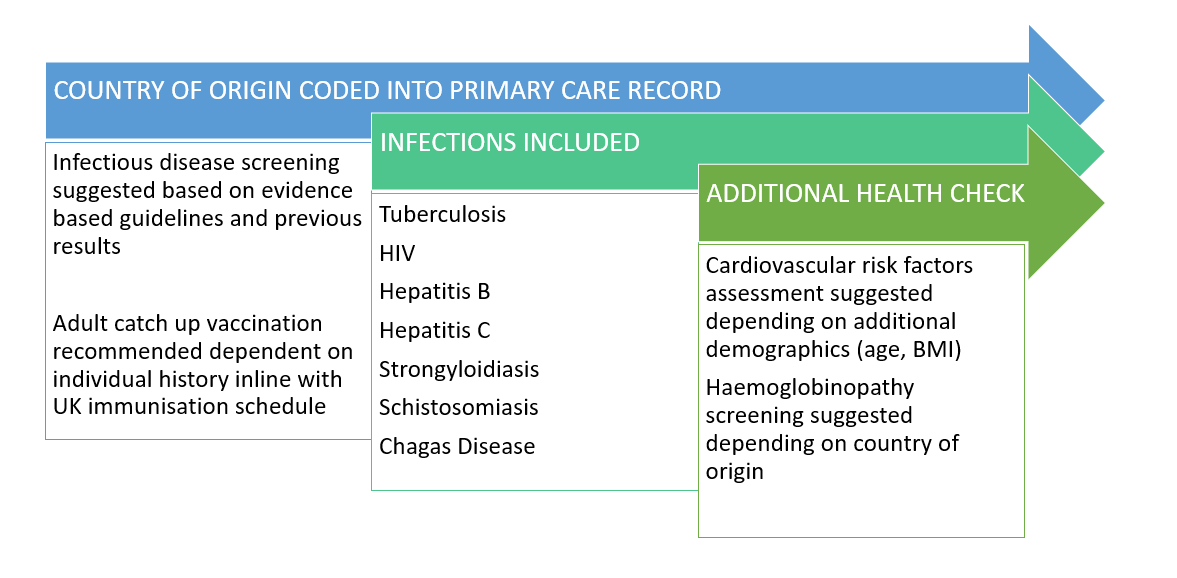
Supplemental Figure 2: Health Catch-UP! Screening and Catch-up Vaccination Prompts**

**
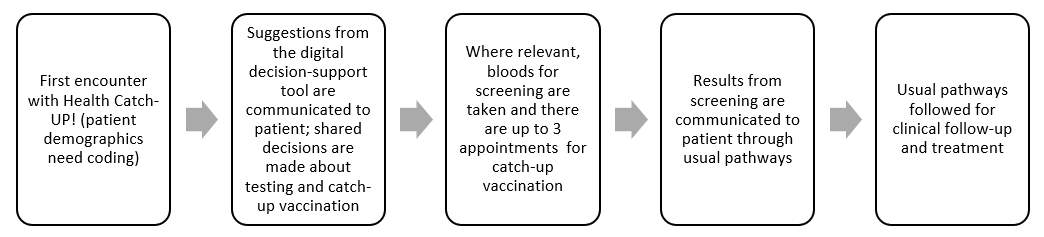
Supplemental Figure 3: Health Catch-Up! Care Pathway**

**Health Catch UP! Demonstration link:** [https://emishealth.vids.io/videos/a49ad1bb1a18e4c72c/health-catch-up-with-requested-edits-mp4](about:blank).
